# Improving LLIN utilization and coverage through an innovative distribution and malaria education model: a pilot study in Okavango Sub-District, Botswana

**DOI:** 10.1186/1475-2875-11-S1-P95

**Published:** 2012-10-15

**Authors:** Simon Chihanga, Allison Tatarsky, HT Masendu, D Ntebela, Tjantilili Mosweunyane, Mpho Motlaleng, Godira Segoea, Justin M Cohen, Mercy Puso, Bosiela Segogo, Tshepo Andina

**Affiliations:** 1 National Malaria Programme, Ministry of Health, Gaborone, Botswana; 2 Clinton Health Access Initiative, Boston, Massachusetts, USA; 3 UNICEF, Gaborone, Botswana; 4 District Health Management Team, Ministry of Health, Okavango Sub-District, Botswana

## Background

The Botswana Malaria Indicator Survey in 2007 indicated remarkably low ITN coverage and usage rates of 9.4% and 5.3%, respectively. To achieve universal coverage of LLINs, optimize uptake, and catalyze Botswana toward its 2015 goal of elimination, Botswana tested a new delivery model to replace its revolving fund scheme of selling subsidized nets to communities at risk.

## Materials and methods

Okavango Sub-District experienced the highest malaria burden in the country (36% of total burden) and was thus chosen as the pilot site. After comprehensive training, sub-district health staff distributed 32,000 LLINs free of charge door-to-door in 2009 and an additional 6,500 LLINs in 2010 and offered hanging assistance with kits of hooks and strings to households. All nets distributed were mapped by household. Both distributions were supported by health education campaigns, including household visits, roadshows, and calendar posters. Two surveys in 2009 and 2010 evaluated pilot outcomes using a structured questionnaire to interview 557 and 362 randomly selected households in Okavango. Findings from the pilot study informed scale up of LLINs throughout Botswana.

## Results

The pilot successfully increased LLIN ownership in Okavango from 13% of households owning at least one ITN in 2007 to 89% of households owning at least one LLIN in 2009 and 94% in 2010. LLIN usage also improved markedly from 5.3% of residents sleeping under an ITN in 2007 to 38% of residents sleeping under an LLIN in 2009 and 46% in 2010. In 2009, 73% of LLINs were immediately hung with the assistance of distributors, and the probability of using an LLIN was 13% higher if households were assisted in hanging the LLINs. Households with a visible poster were 26% more likely to use an LLIN, and subsequent health education visits were significantly associated with higher usage (p=0.0005) - after no visits, 64% of nets were used while after three visits, 83% of nets were used. The majority of respondents received their malaria messages from LLIN distributors, health talks at clinics, and visits by health educators, indicating the critical role of door-to-door visits in increasing awareness about malaria and LLINs.

An intra-household analysis found that smaller households generally owned enough LLINs to cover all household members but larger households did not receive enough nets, indicating unequal distribution of LLINs. Moreover, in households with 100% coverage, 67% of residents used an LLIN in 2010 - a substantially higher rate than the district-wide rate of 46% that does not take into account whether households actually owned enough nets to use them.

Clinical malaria cases declined from 6,446 in 2008 to 22 in 2011 and confirmed cases declined from 183 to 15. Alongside ongoing IRS, more robust implementation of parasitological diagnosis, and other interventions, the pilot was associated with a substantial decline in malaria cases in Okavango.

## Conclusion

Overall, the pilot achieved mass free distribution to over 64,000 individuals and significantly increased LLIN ownership and usage. Lessons learned from this pilot informed distribution of additional LLINs to all at risk areas in Botswana and this model was brought to scale successfully.

